# Functional and structural insights into HCMV terminase accessory proteins pUL77 and pUL93

**DOI:** 10.1128/jvi.01173-25

**Published:** 2025-09-16

**Authors:** C. Gourin, F. Di Meo, C. Delmon, S. Alain, S. Hantz

**Affiliations:** 1 Inserm, CHU Limoges, University of Limoges, RESINFIT, U1092 54999, Limoges, France; 2 Inserm U1248 Pharmacology & Transplantation, Univ. Limoges, Limoges, France; 3 Inserm US042/CNRS UAR 2015 Integrative Biology Health Chemistry & Environment, Univ. Limoges, Limoges, France; 4 CHU Limoges, Laboratoire de Bactériologie-Virologie-Hygiène, National Reference Center for Herpesviruses (NRCHV), Limoges, France; University of Virginia, Charlottesville, Virginia, USA

**Keywords:** human cytomegalovirus, CVSC, pUL77, pUL93, terminase complex, letermovir

## Abstract

Human cytomegalovirus (HCMV) is a significant cause of morbidity and mortality in immunocompromised and congenitally infected patients. Current antiviral therapies, primarily targeting the viral DNA polymerase, are limited by toxicity and resistance, underscoring the need for novel therapeutic strategies. Letermovir (LTV), which targets the viral terminase complex (pUL56-pUL89-pUL51), has improved prophylaxis in transplant recipients, but resistance mutations have already emerged. This complex is responsible for cleaving and packaging the viral genome into neo-formed capsids in close interaction with several other proteins. This study focuses on two essential terminase-associated proteins, pUL77 and pUL93, investigating their natural polymorphism, mutations arising under LTV prophylaxis, and the functional relevance of their putative nuclear localization signals (NLS). Sequence alignments across 18 herpesviruses revealed conserved and variable regions in both proteins. New mutations emerging in patients treated with LTV did not confer resistance, but the pUL77 R43C mutation, when present with pUL56 C325F, significantly reduces LTV resistance compared to C325F alone. In addition, nuclear localization motifs were assessed in the viral replication context and in autonomous cell production after plasmid transfection and revealed motifs essential for viral replication: _43_RVRKRYLRQ_55_ and _225_PRWKRV_231_ in pUL77 and _505_RDRRGRLRR_513_ in pUL93. Finally, AI-based modeling provided some insights into the functions of these motifs for both proteins, notably in interaction with other viral proteins. Collectively, these data provide a wealth of information about putative functional sites in pUL77 and pUL93 that could be new targets for anti-HCMV strategies such as inhibitory peptides.

**IMPORTANCE:**

Human cytomegalovirus (HCMV) remains a significant health concern, particularly for immunocompromised individuals and in cases of congenital infection. The emergence of resistance to current antivirals targeting viral polymerase has necessitated the development of novel therapeutic approaches. Letermovir, which targets the HCMV terminase complex, represents a promising alternative. However, a deeper understanding of the virus’s structural components and their interactions is crucial for identifying new potential drug targets. Here, we focused on two proteins, pUL77 and pUL93, which are associated with the viral capsid and interact with the terminase complex. By analyzing their polymorphism, structure, and functional motifs, we identified essential nuclear localization motifs and conserved regions in these proteins, providing valuable insights for the development of innovative anti-HCMV strategies, such as inhibitory peptides, which could complement existing treatments and address the ongoing challenge of antiviral resistance.

## INTRODUCTION

Human cytomegalovirus (HCMV) is responsible for higher morbidity and mortality rates in immunocompromised patients, such as transplant recipients and AIDS patients (1). HCMV infection is also the main cause of congenital viral infection, responsible for severe malformations (2). Most anti-CMV drugs targeted only the viral DNA polymerase pUL54. Although their established efficacy, their use remains limited not only owing to dose-limiting toxicity but also to the emergence of resistance leading to therapeutic failure. Targeting a new stage in the viral replication cycle, letermovir (LTV) was demonstrated to be effective as a prophylactic treatment against HCMV in hematopoietic stem cell recipients and approved by the US and European Medicines Agencies (3). More recently, the efficacy in prophylaxis was also demonstrated in kidney recipients (4). LTV was shown to inhibit the viral terminase complex made of three proteins, namely pUL56, pUL89, and pUL51 (5). Nevertheless, *in vitro* and *in vivo* resistance mutations have already been identified (6–10). Therefore, there is a critical need for new therapeutic strategies targeting essential viral proteins.

The terminase complex (pUL56, pUL89, and pUL51) is responsible for cleaving DNA into unit-length genomes and packaging them into neoformed capsids (5). The large pUL56 subunit and the small subunit pUL89 contain the functional sites required for DNA cleavage (11, 12). Noteworthy, three additional proteins were shown to be involved in this event, namely, pUL52, pUL77, and pUL93 (13, 14). pUL77 and pUL93 are, respectively, encoded by the *UL77* and *UL93* genes, which are found on the unique long region of the HCMV genome. pUL77 and pUL93 are, respectively, 642 and 593 amino acid proteins with molecular masses of 71 and 68 kDa. They were shown to be structural proteins associated with *Herpesviridae* capsids (15, 16). pUL77 and pUL93 proteins are well conserved along the Herpesvirus family, and their homologs in herpes simplex type 1 (HSV-1) are, respectively, pUL25 and pUL17 (17, 18).

pUL77 contains a putative pyruvoyl decarboxylase site _613_TLGSSLFN_620_ in the C-terminal domain (19). The first hundred amino acids of pUL77 (residues 1 to 100) adopt a spiral conformation (CCM) responsible for the protein homodimeric conformation (15). The first 48 amino acids of the CCM were proposed to interact with the so-called major capsid protein (MCP), as well as the DNA during the DNA-packaging step (15). Köppen-Rung et al. suggested the existence of two motifs in pUL77 as nuclear localization signals (NLS) in the N-terminal domain, namely _55_RVRKRYLRQ_63_ and _219_YYRLKRG(LYTQ)PRWKRV_231_ (16). However, their study explained that these motifs were not responsible for nuclear localization by themselves. Furthermore, pUL77 was shown to also interact with pUL48, which is involved in the tegument structure (20). pUL93 has been identified as a crucial partner for the correct subnuclear localization of pUL77 (13). pUL93 interacts with the so-called nuclear egress complex (NEC) consisting of pUL53, pUL50, and pUL97 (21). Two putative NLSs were also identified in the pUL93 sequence: _177_KRDRQHQLATATNHRRR_193_ and _442_RARRQ_447_, but have never been studied (21). Moreover, Borst et al. demonstrated that only empty B-capsids were found in the absence of both pUL77 and pUL93 proteins (22). Interestingly, the three-dimensional structures of pUL77 and pUL93 were partially resolved by means of cryogenic electron microscopy within the vertex-specific component complex (CVSC) (23). A complementary study based on artificial intelligence has completed the models of the three-dimensional structure of the HCMV tegument, including CVSC proteins as pUL77 and pUL93 (24).

In the present study, we investigated pUL77 and pUL93 proteins considering (i) their natural polymorphism and (ii) those triggered under LTV selective pressure. New mutations were described and assessed in the LTV plaque assay alone or in combination with *UL56* resistance mutations. Additionally, putative NLSs of pUL77 and pUL93 were identified and monitored to provide robust hints regarding their functional domains. Such findings are supported by using structural approaches and the comparison with their respective HSV-1 homologs, namely pUL25 and pUL17 for pUL77 and pUL93, respectively.

## RESULTS

### Determination of conserved domains and polymorphism analysis

**Fig 1:**
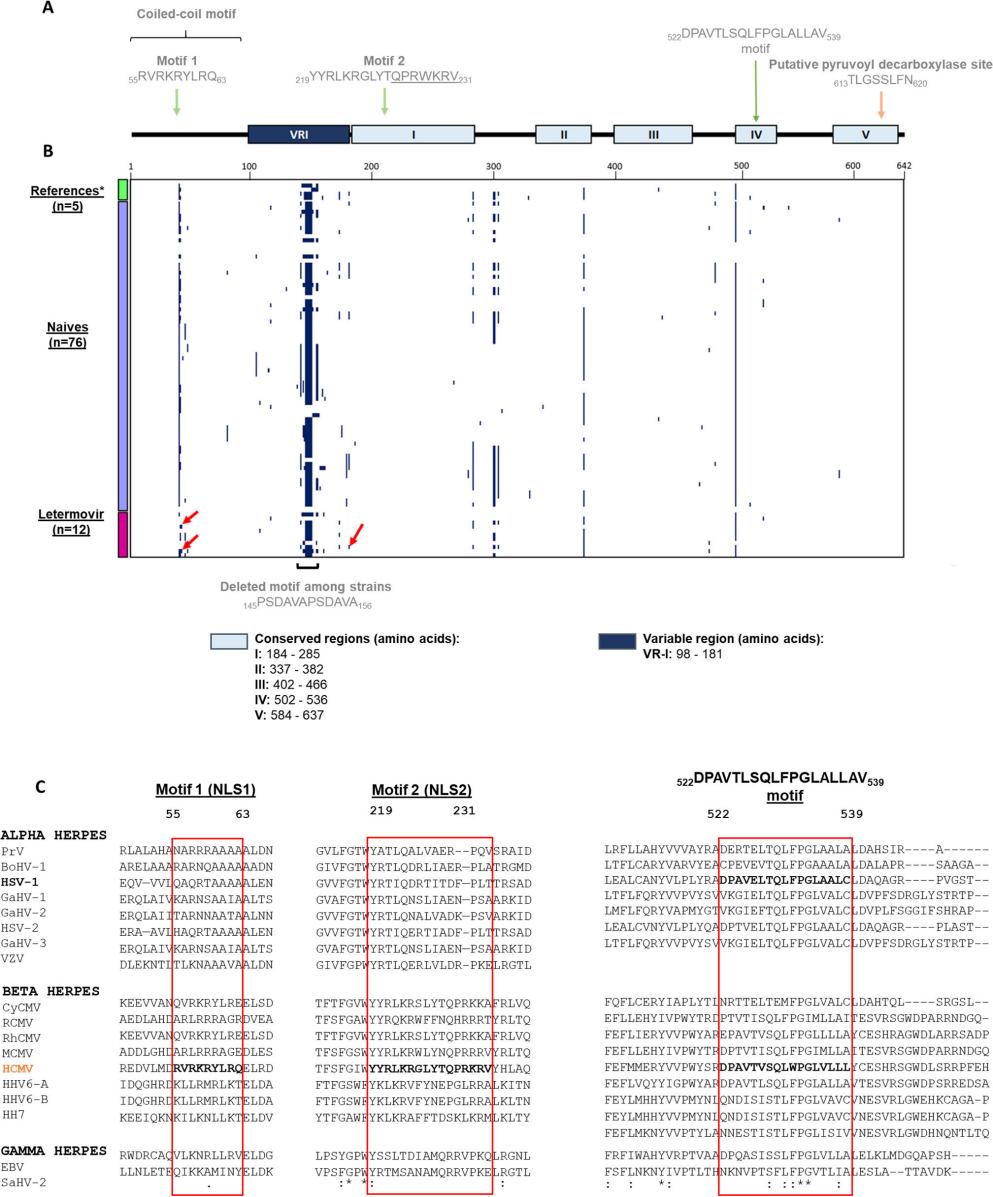
Conserved regions and polymorphism of HCMV pUL77. (**A**) pUL77 is composed of one variable (dark blue; VRI) and five conserved regions (light blue; I–V). Two motifs similar to putative NLSs are shown by green arrows in amino acid positions 55 to 63 and 219 to 231. Those were studied by Köppen-Rung et al. (16). A putative pyruvoyl decarboxylase site cited in the literature is also shown by a green arrow in acid amino positions 613 to 620. The coiled-coil motif of the protein concerns the first one hundred amino acids of pUL77. (**B**) Polymorphism representation of pUL77 through a binary heatmap according to strains and amino acid positions in pUL77. The three groups studied (reference strains [*n* = 5]; *AD169, Toledo, Towne, Merlin, Davis strains), naïve strains (*n* = 76), and strains from patients treated with LTV (*n* = 12) are represented by green, mauve, and pink boxes, respectively, on the y-axis. The absence of mutation is represented in white, and mutations in dark blue. Red arrows show new mutations under letermovir selective pressure. VR, variable region; NLS, nuclear localization signal. (**C**) Sequence alignment of the pUL77 NLS1, NLS2, and the _522_DPAVTLSQLFPGLALLAV_539_ motif from 18 herpesviruses. Sequences of interest are in bold.

**Fig 2:**
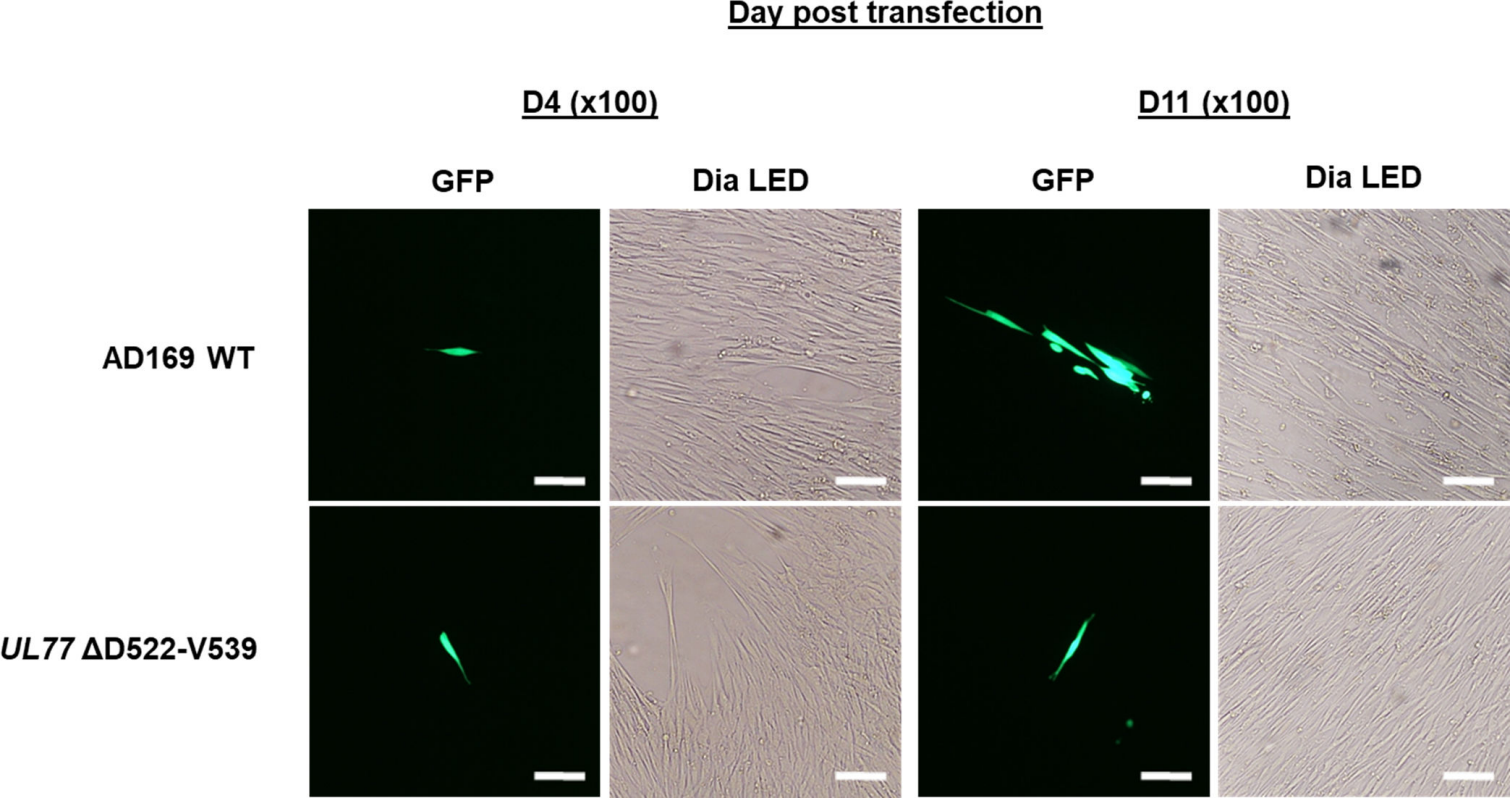
Transient transfection of HCMV BAC mutant pUL77 ΔD522-V539. Green fluorescent protein (GFP) and DIA LED (optical light) observation after 4 days (D4) and 11 days (D11) post-transfection for mutant pUL77 ΔD522-V539 compared to the AD169 wild-type strain. Magnification ×100. Scale bar: 100 µm.

The homologs from 18 herpesviruses (Table S1) were considered for sequence alignments: 16 amino acids were identical, 38 were strongly similar, and 15 were weakly similar. Various gaps were observed in the pUL77 N-terminal region (residues 98 to 181), suggesting the existence of a variable region (VR-I, Fig. 1A and B). Sequence alignments also revealed the existence of five conserved regions, namely region I (residues 184 to 285), region II (residues 337 to 382), region III (residues 402 to 466), region IV (residues 502 to 536), and region V (residues 584 to 637) (Fig. 1A and B). By comparing the sequence of pUL77 with that of 18 homologs, the putative pUL77 NLS1 and NLS2 are not conserved among other herpesviruses. In contrast, another motif from residue 522 to 539 (_522_DPAVTLSQLFPGLALLAV_539_) located in region IV is relatively conserved among the 18 herpesviruses (Fig. 1C). This domain was deleted in the HCMV-bacterial artificial chromosome (BAC). Eleven days after transfection in MRC-5 cells, no replication of the recombinant virus was observed (Fig. 2).

**Fig 3:**
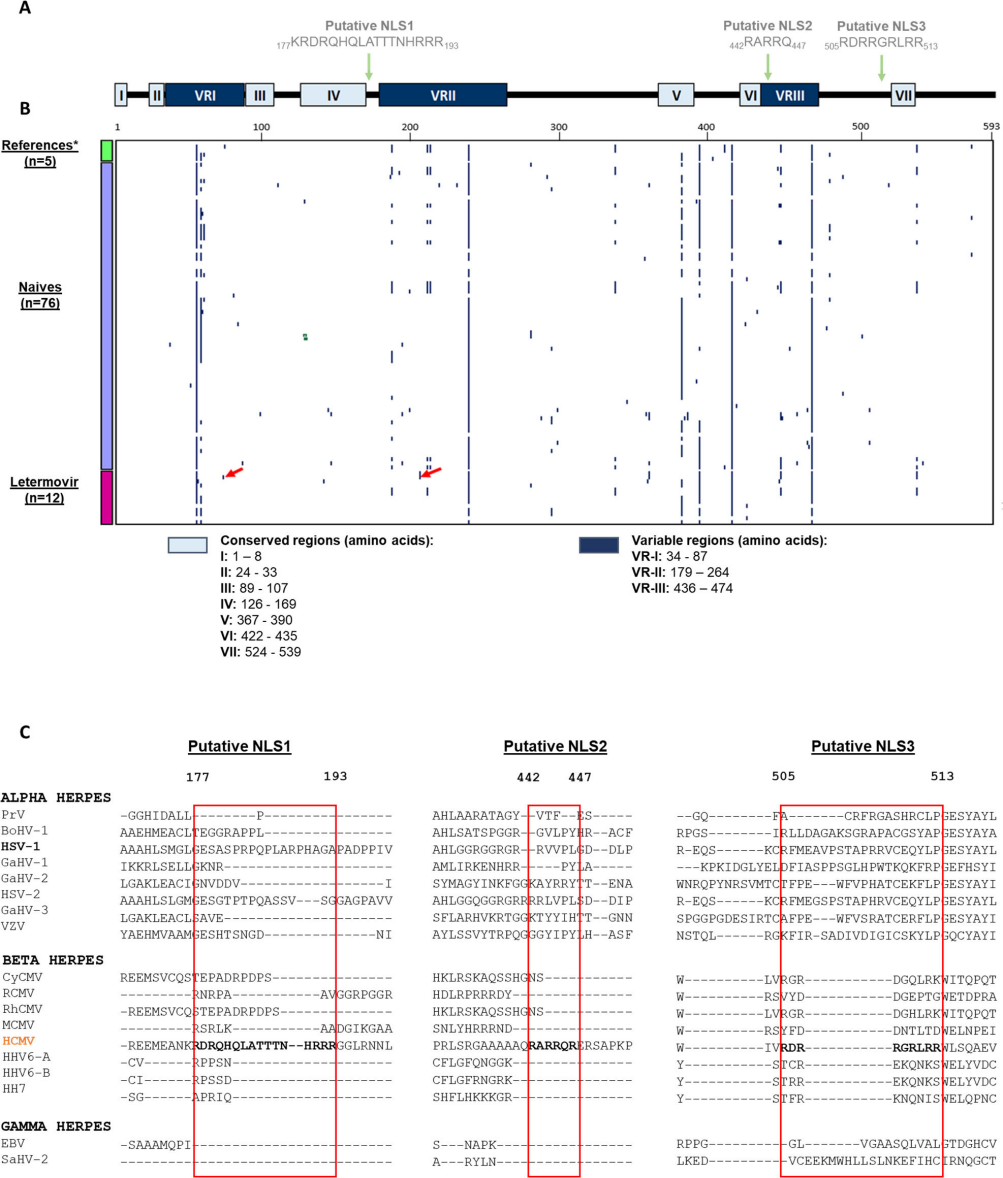
Conserved regions and polymorphism of HCMV pUL93. (**A**) pUL93 is composed of three variable (dark blue; VRI) and seven conserved regions (light blue; I–VII). Three putative NLSs are shown by green arrows in amino acid positions 177 to 193, 442 to 447, and 505 to 513. The coiled-coil motif of the protein concerns the one first hundred amino acids of pUL93. (**B**) Polymorphism representation of pUL93 through a binary heatmap according to strains and amino acid position in pUL93. The three groups studied (reference strains [*n* = 5]; *AD169, Toledo, Towne, Merlin, Davis strains), naïve strains (*n* = 76), and strains from patients treated with letermovir (*n* = 12) are represented by green, mauve, and pink boxes, respectively, on the y-axis. The absence of mutation is represented in white, and mutations in dark blue. Red arrows show new mutations under letermovir selective pressure. VR, variable regions; NLS, nuclear localization signal. (**C**) Sequences alignment of the pUL93 NLS1, 2, and 3 from 18 herpesviruses. HCMV sequences of interest are in bold.

Similar analyses were conducted with pUL93 (Table S2): 10 amino acids were identical, five were strongly similar, and six were weakly similar. In contrast with pUL77, numerous regions with gaps were observed, suggesting at least three variable regions, namely VR-I (from residue 34 to 87), VR-II (from residue 179 to 264), and VR-III (from residue 436 to 474). Sequence alignment also revealed seven conserved regions located either in the N- and C-terminal domains (Fig. 3A). Polymorphism analysis of pUL93 is in agreement with these results, most of the mutations being in variable regions (Fig. 3B). The putative pUL93 NLSs were all located in non-conserved regions of the protein. Furthermore, these NLSs were not observed in the other 18 herpesviruses, suggesting specific roles for HCMV (Fig. 3C).

Reference strain sequences of AD169, Towne, Toledo, Merlin, and Davis were identical to those from the GenBank database (accession numbers FJ527563, JX512198, GU937742, FJ1616285.1, and NC_006273) for pUL77 and pUL93. Among the 76 naive strains, we reported 51 and 63 amino acid polymorphisms distributed over pUL77 and pUL93, respectively. Twenty and 18 polymorphisms were also present in reference strains for each protein, respectively. The average identities of the HCMV isolates were 98.6% and 99.2% for pUL77 and pUL93, respectively. Analysis of polymorphism distribution across pUL77 revealed a region with a high number of mutations, as well as the deletion of the repetitive _145_PSDAVAPSDAVA_156_ motif, observed in most of the reference and clinical HCMV strains. This is consistent with sequence alignment analyses for which the variable region was annotated as pUL77 VR-I. Interestingly, this motif was not found among other herpesviruses (Fig. 1A and B). Likewise, polymorphism analyses revealed a high sequence variability in VR-I, VR-II, and VR-III of pUL93 (Fig. 3A and B).

Interestingly, while considering the 12 sequences of HCMV strains detected in LTV-treated patients, 26 mutations were found in pUL77; 15 and 9 being, respectively, either shared with reference strains or naive strains. However, we observed two new mutations: R43C and A161T (see red arrows in Fig. 1B). R43C was assigned to the non-conserved region belonging to the coiled-coil motif of the protein, while A161T was located in the pUL77 VR-I. For pUL93, 18 mutations were found in HCMV strains detected in LTV-treated patients, of which 13 were identical to reference strains and three to naive strains. Again, we observed two new mutations located either in pUL93 VR-I or VR-II: E73G and R206H (see red arrows in Fig. 3B). Mutation pUL77 R43C was associated with C325F in strain 5 and was combined with pUL77 A161T and pUL56 C325Y in patient 11. pUL93 E73G and R206H were associated with pUL56 V236M in clinical strain 2. An overview of clinical mutations in pUL56, pUL77, and pUL93 is available in Table S3.

### Assessment of new mutations

**Fig 4:**
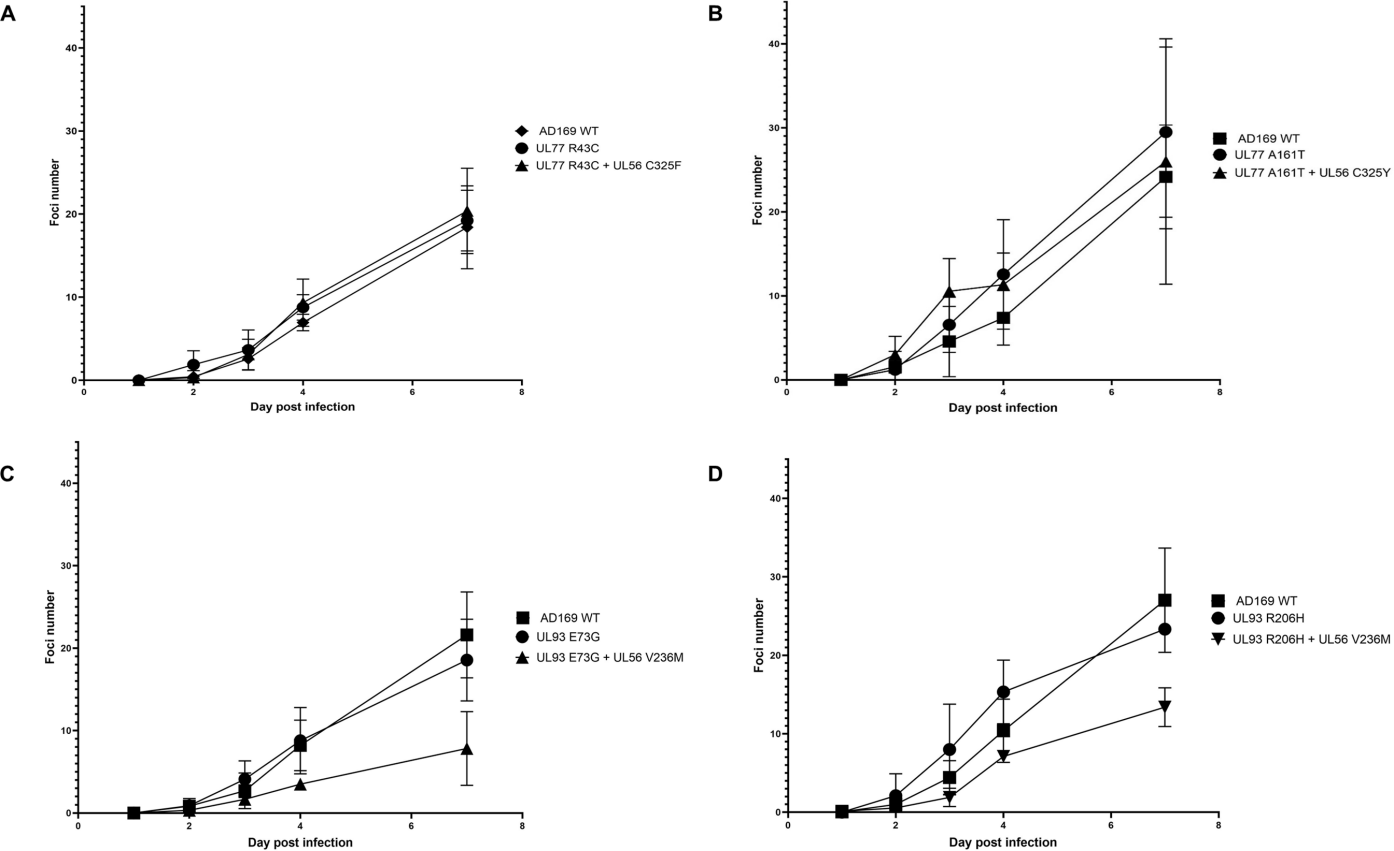
Growth curve analysis of recombinant HCMV-BAC of pUL77 and pUL93. Viral foci were counted during 7 days according to GFP fluorescence. All data were generated by three independent experiments (created with GraphPad Prism 8.4.3). (**A**) Growth curves of pUL77 R43C mutant and pUL56 C325F double mutant. (**B**) Growth curves of pUL77 A161T mutants and UL56 C325Y combined mutation. (**C**) Growth curves of pUL93 E73G and pUL56 V236M combined mutation. (**D**) Growth curves of pUL93 R206H mutant and pUL56 V236M combined mutations.

Even though amino acids identified from pUL77 and pUL93 are not conserved, we investigated their potential impact by introducing these mutations in HCMV-BAC and transfecting it into MRC-5 human embryonic fibroblasts. Plaque assays with LTV exhibited that standalone mutations do not confer LTV resistance to recombinant viruses as compared with AD169 (Table 1). These mutations were also combined with V236M, C325F, or C325Y mutations in pUL56, known to be associated with LTV resistance, as they were detected by resistance genotyping in the clinical follow-up of these patients. Plaque tests with the LTV of the C325F/R43C mutant appeared to significantly reduce the level of resistance compared with the C325F mutant alone (*P* = 0.0261). For the other mutants, no significant change in EC_50_ of strains with the UL56 mutations was observed, independent of the presence of the other pUL77/pUL93 mutations (Table 1). Finally, cell growth was also investigated considering these mutations by means of green fluorescent protein (GFP) counts, using the AD169 strain as a reference. In line with plaque assays, these new mutations were not associated with a modulation of viral fitness (Fig. 4).

**TABLE 1:** *In vitro* effective concentration 50% for letermovir (EC_50_) of the UL56, UL77, and UL93 recombinant HCMV-BAC harboring the different mutations found in strains from LTV treated patients^*c*^

Strain	Gene mutation			LTV EC_50_ (±SD^*a*^)	Ratio^*b*^	*P* value
	*UL56*	*UL77*	*UL93*			
AD169	–^*d*^	–	–	2.31 (±0.59) nM		
AD169	–	R43C	–	3.15 (±0.71) nM	1.36	0.3336
AD169	–	A161T	–	2.82 (±0.26) nM	1.22	0.5162
AD169	–	–	E73G	2.45 (±0.19) nM	1.06	0.8617
AD169	–	–	R206H	2.57 (±0.44) nM	1.11	0.7501
AD169	V236M	–	–	143.42 (±23.17) nM	**62.09**	
AD169	V236M	–	E73G	134.89 (±6.32) nM	**58.39**	0.7736
AD169	V236M	–	R206H	196.18 (±14.28) nM	**84.92**	0.1685
AD169	C325F	–	–	30.49 (±1.06) µM	**13,198.85**	
AD169	C325F	R43C	–	21.72 (±1.93) µM	**9,402.16**	0.0261
AD169	C325Y	–	–	33.57 (±2.05) µM	**14,532.47**	
AD169	C325Y	A161T	–	31.66 (±2.34) µM	**13,706.93**	0.6609

^*a*^ Standard deviation of EC_50_ values from three assays.

^*b*^ Ratio of EC_50_ values to that of wild-type control strain AD169.

^*c*^ Bold values, significant resistance. Underlined values, EC_50_ significant change of new mutants compared to already described mutants or AD169 strain.

^*d*^ –, no mutation.

### Study of putative nuclear localization signals alone and in a viral replication context

**Fig 5:**
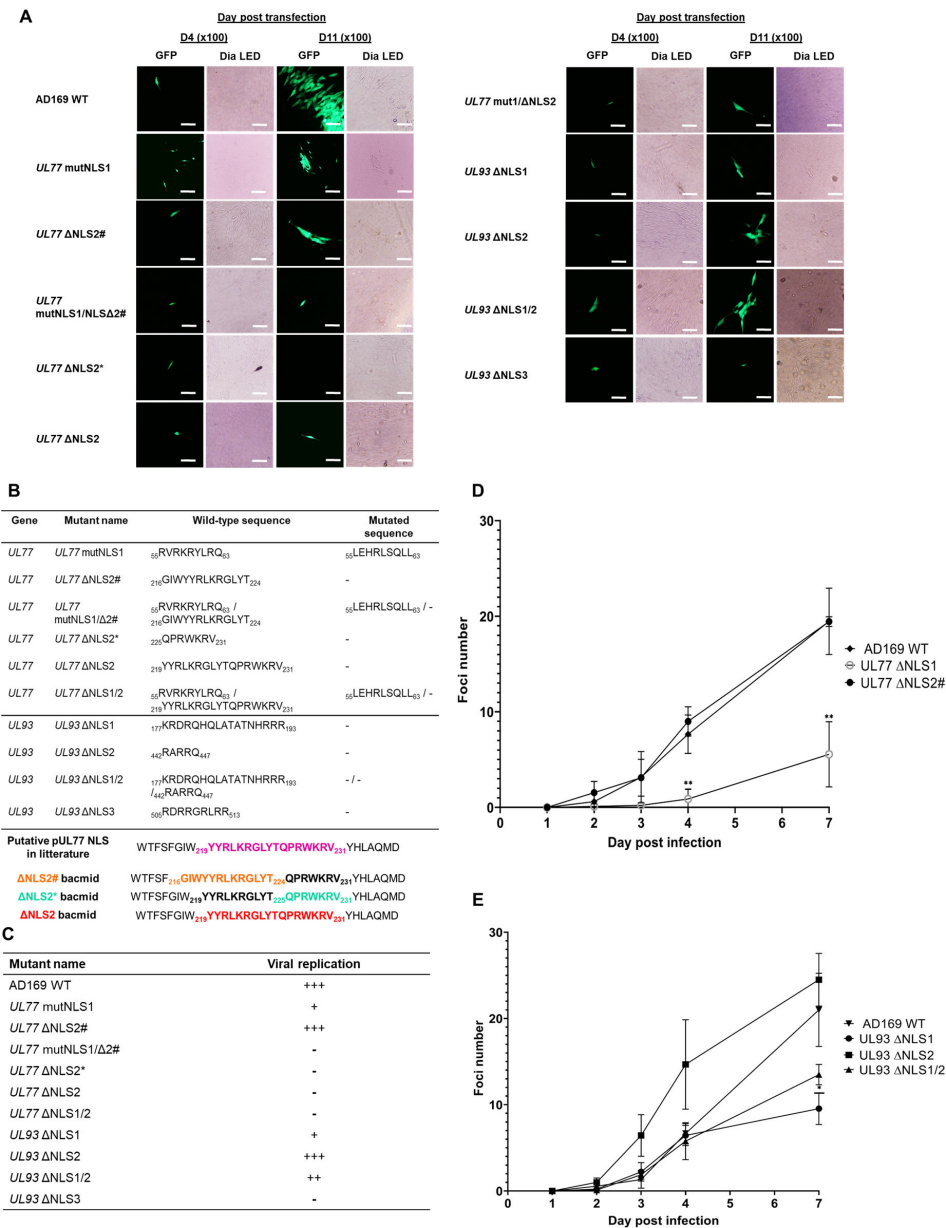
Study of putative NLSs in HCMV pUL77 and pUL93. (**A**) GFP fluorescence and DIA LED (optical light) observation after 4 days (D4) and 11 days (D11) post-transfection for NLSs recombinant HCMV-BAC compared to the AD169 wild-type strain. For pUL77, only the mutNLS1 and ΔNLS2# led to viral replication with observation of viral foci. For pUL93, only the ΔNLS3 failed to replicate. Magnification ×100. Scale bar: 100 µm. (**B**) Summary of pUL77 and pUL93 NLSs recombinant viruses with their names, wild-type amino acid sequences, and mutated sequences. (**C**). Non-statistical summary of the fitness of NLSs mutant HCMV-BAC with AD169 as 100% control according to foci counts. (**D**) Impact of HCMV-BAC-*UL77* mutants on growth in cell culture (MRC-5 fibroblasts) during 7 days (x-axis). (**E**) Impact of HCMV-BAC-*UL93* mutants on growth in cell culture (MRC-5 fibroblasts) during 7 days (x-axis). Viral foci were counted according to GFP fluorescence and represent the y-axis. The AD169 wild-type strain was used as a reference. Created with GraphPad Prism 8.4.3. D4, day 4; D11, day 11; WT, wild type; NLS, nuclear localization signal; Δ, deleted; mut, mutated.

Putative nuclear localization signals reported in the literature were either mutated or deleted in HCMV-BAC GFP and transfected into MRC-5 cells to define their role. Standalone mutations were carried out, and multiple mutation combinations were also performed as reported in Fig. 5. It is important to note that pUL77 NLS1 was only mutated and not deleted due to its overlap region with the ORF region of the pUL76 protein. Moreover, to ensure that pUL77 NLS2 was a bipartite functional domain as described in the literature, three mutants were constructed: NLS2#, NLS2*, and NLS2. Indeed, the second whole putative NLS of pUL77 (NLS2) cited in the literature was _219_YYRLKRG(LYTQ)PRWKRV_231_ and was supposed to be bipartite (16). So we constructed two additional mutants, NLS2# and NLS2*, harboring deletions of the sequences _216_GIWYYRLKRGLYT_224_ and _225_QPRWKRV_231_, respectively. This analysis allowed us to assess the bipartite character of the putative NLS2 (Fig. 5A). Growth fitness assays were carried out using the AD169 strain as a reference. In the fitness analysis, the standalone pUL77 NLS1 mutation had a significantly attenuated growth at D4 and D7 (*P* values = 0.001) (Fig. 5A and D). Likewise, the standalone deletion of the _225_QPRWKRV_231_ motif of pUL77 NLS2 (NLS2*) led to the absence of replication. For pUL77 NLS2# deletion, no inhibition of viral replication was observed (*P* = 0.93) (Fig. 5A and D). The pUL93 ΔNLS1 mutant showed a significant reduction in fitness at D7 (*P* value = 0.02), but the pUL93 ΔNLS2 and ΔNLS1/2 mutants did not (*P* = 0.44 and *P* = 0.14, respectively) (Fig. 5E). Using Geneious software, we obtained isoelectric point (pI = 12.80) of pUL93 _505_RDRRGRLRR_513_ suggesting a third putative NLS in pUL93 (*UL93* NLS3) located in the C-terminal region (Fig. 1A and 5B). No cytopathic effects were observed after transfection of the recombinant HCMV-BAC pUL93 ΔNLS3 in MRC-5 cells (Fig. 5A and C).

### Study of putative nuclear localization signal in HEK293T cells

**Fig 6:**
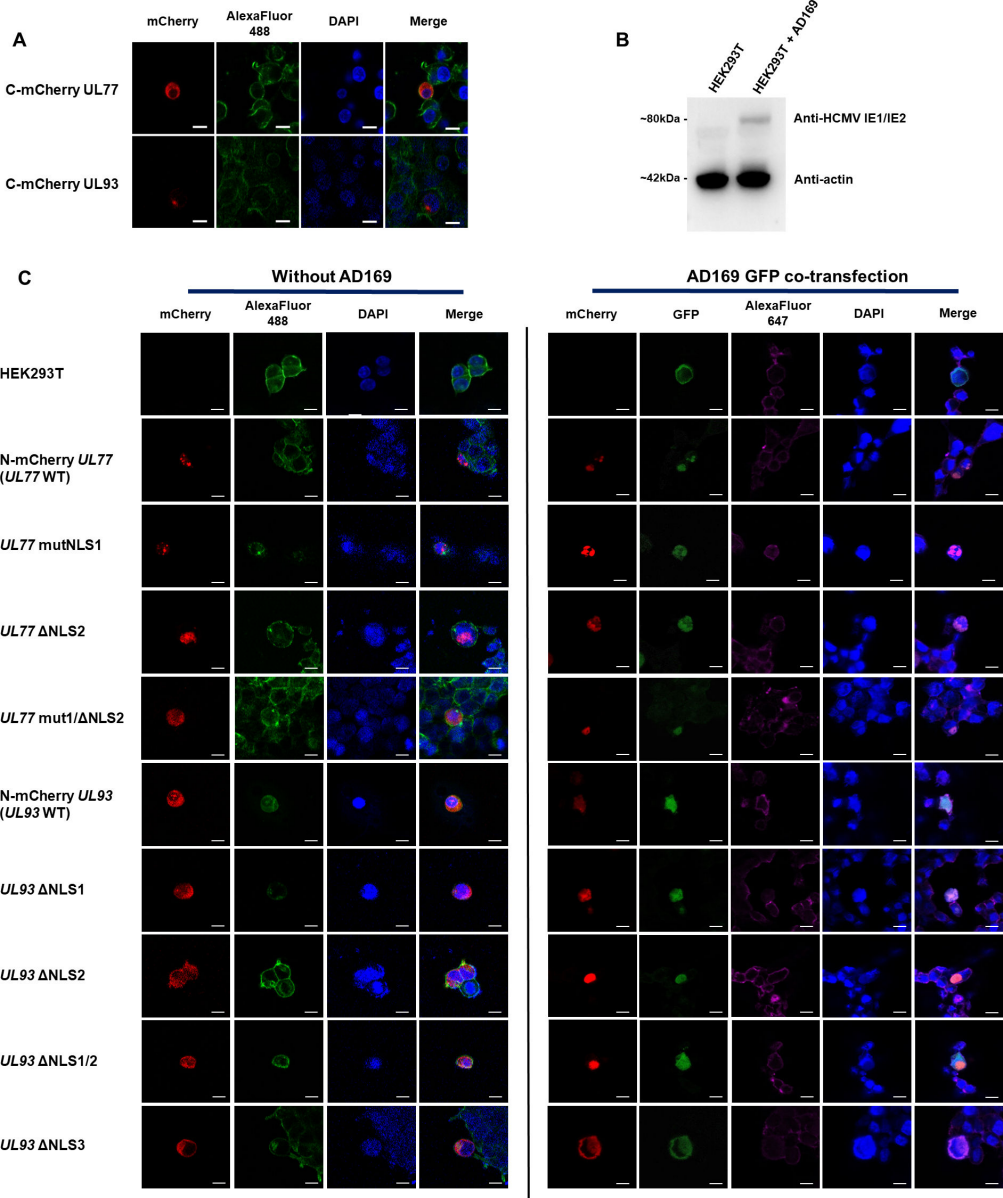
Fluorescence microscopy analysis of nuclear crossing events of pUL77 and pUL93. (**A**) Fluorescence confocal microscopy images of cloned pUL77 and pUL93 with mCherry in the C-terminal part. C-terminal cloning results show the inhibition of wild-type pUL77 and pUL93 passage through the nucleus. (**B**) Western blot analysis of the production of AD169 by HEK293T cells using anti-cytomegalovirus IE1/IE2 antibody. (**C**) mCherry mutant proteins localization in HEK293T using AlexaFluor 488 phalloidin stain and 4′,6-diamidino-2-phenylindole (DAPI) counterstain to observe actin and nucleus of cells, respectively. The merging of three fluorescent signals gives the protein emplacement according to cell morphology. Same experiment with GFP-HCMV-BAC (AD169) co-transfection. AlexaFluor 647 stained cell membranes. mCherry: red fluorescence; DAPI: blue fluorescence in nuclei; AlexaFluor 488: green fluorescence of actin filaments; AlexaFluor 647: far-red (magenta) fluorescence of cell membranes; GFP: green fluorescence of HCMV. Magnification ×630 with oil immersion. Scale bar: 10 µm.

To assess the cellular localization of wild-type or mutated pUL77 and pUL93, mCherry-protein recombinants were built and monitored by confocal microscopy. mCherry was appended to pUL77 and pUL93 N-terminal domains in order to ensure protein functions (Fig. 6A and B). The same experiment was performed with co-transfection of HCMV-BAC. In this latest experiment, only cells co-expressing GFP and mCherry were analyzed. HCMV AD169 production was assessed by western blot using IE1/IE2 protein detection (Fig. 6B).

**Fig 7:**
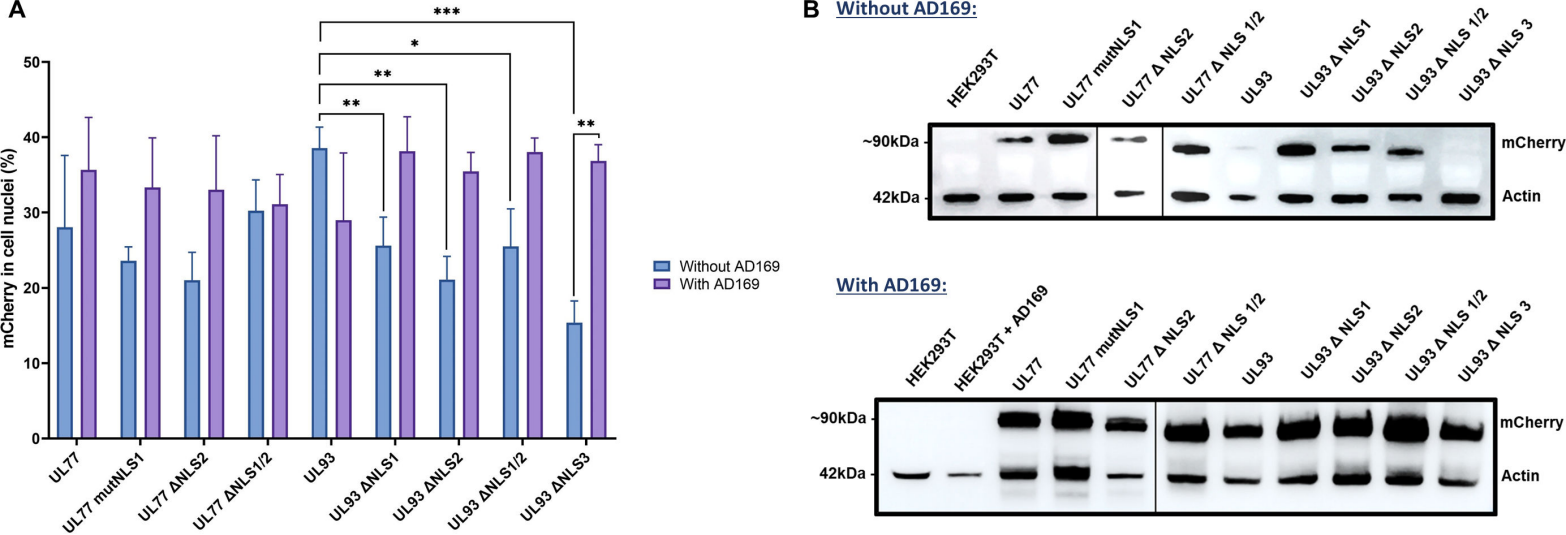
Production and study of putative pUL77 and pUL93 NLSs in HEK293T with and without viral machinery. (**A**) Percentage of red fluorescence in cell nuclei according to DAPI. (**B**) Western blot analysis for the production of mCherry pUL77 and pUL93 fused clones in HEK293T with mCherry monoclonal antibody and HRP-anti-rabbit polyclonal antibody. The reference protein was actin and was revealed using an HRP anti-actin antibody. Chemiblot exposure for 10 s. *P < 0.05, **P < 0.01, ***P < 0.001.

The percentage of mCherry in cell nuclei was determined as a function of fluorescence intensity and number of red pixels (see Fig. 6 and 7). Wild-type proteins were all observed in cell nuclei, suggesting the crossing of the nuclear membrane (Fig. 6C). Standalone or combined modifications of pUL77 NLSs did not significantly affect pUL77 entry into cell nuclei. Interestingly, the standalone deletion of pUL93 NLS1 or NLS2 and the combined deletion of NLS1/NLS2 led to a significant decrease of pUL93 signal in the cell nucleus (*P* = 0.009; *P* = 0.002; *P* = 0.02, respectively). The deletion of pUL93 NLS3 alone impacted the nuclear crossing of the protein (*P* < 0.001). However, the co-transfection of AD169 significantly restored the function of pUL93 to entry into the cell nucleus (*P* = 0.0012) (Fig. 6C and 7A).

Western blot assays were conducted to verify the construction of mCherry-fused clones and allowed us to see that proteins were produced at different levels by normalizing them against the actin control protein. The addition of AD169 greatly increased the production of all clones (Fig. 7B).

### Structural investigation of HCMV pUL77 and pUL93

**Fig 8:**
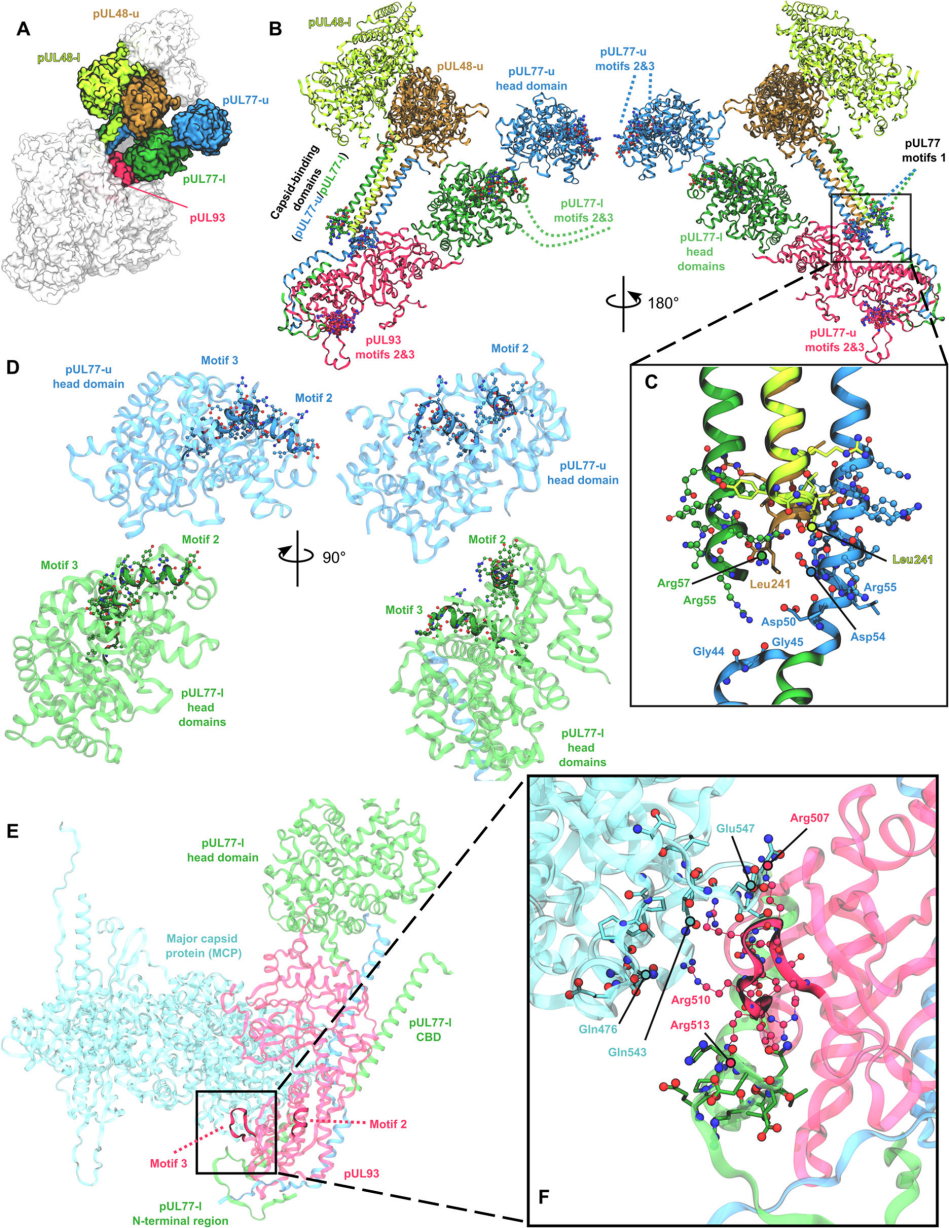
Structural overview of HCMV pUL77 and pUL93 motifs. (**A**) Overall structure of a monomer HCMV portal vertex adopting virion configuration 1 from PDB ID 8TEP. Dimers of pUL77 (upper pUL77-u, blue; lower pUL77-l, green) and pUL48 (upper pUL48-u, brown; lower pUL48-l, yellow) and pUL93 (red) are highlighted. For the sake of readability, the color code will be kept in the figure. (**B**) Secondary structures of pUL48 dimer, pUL77 dimer, and pUL93, and localization of pUL77 and pUL93 motifs (van der Waals representation), notably in pUL77 head capsid binding domains. pUL93 motif 1 is not shown since it was not resolved in the cryo-EM structure. (**C**) Zoom of capsid binding domain highlighting key residues involved in salt-bridge and H-bond network maintaining interactions between pUL48 and pUL77 dimers. (**D**) Structural localization of motifs 2 and 3 in the pUL77-u and pUL77-l head domains. (**E**) Structural localization of motifs 2 and 3 in pUL93, highlighting the potential role of motif 3 at the interface with the HCMV MCP (cyan) and pUL77-l N-terminal region. (**F**) Zoom of the interface between pUL93, pUL77-l N-terminal region, and MCP, highlighting pUL93 motif 3 and key residues likely involved in non-covalent interactions.

Taking advantage of the recent resolution of the HCMV portal vertex in several configurations (24), we identified pUL77 and pUL93 motifs within the structure resolved in the virion configuration 1 (PDB ID: 8TEP), representing the post-nuclear translocation state with binding to pUL48 (Fig. 8A). Notably, the portal vertex is a pentameric structure, where each subunit is composed of two pUL77 molecules (pUL77-l and pUL77-u), two pUL48 molecules (pUL48-u and pUL48-l), and a single pUL93 molecule, all associated with other capsid proteins (Fig. 8A) (24). Three motifs within pUL77 were resolved in the cryo-EM structure, with NLS 1 located in the capsid-binding domain (CBD). This domain consists of four α-helices formed by the N-terminal regions of pUL77-l and pUL77-u, in association with the C-terminal regions of pUL48-u and pUL48-l (Fig. 8B), consistent with previous reports (23, 24). Remarkably, NLS 1 is rich in cationic residues (e.g., Arg57 and Arg55), which form strong electrostatic interactions with the carboxylate groups of the C-terminal leucine residues of pUL48-u and pUL48-l (Fig. 8C). These residues are also in close proximity to anionic residues in pUL77-u (Asp50 and Asp54), suggesting the formation of a robust salt-bridge network critical for maintaining the CBD structure. Additionally, the carbonyl groups of Gly44 and Gly45 were observed within 5 Å of the coil region of the helix-coil-helix configuration in the N-terminal domain of pUL77-l, indicating a weaker hydrogen bond network in this region. NLS 2 and motif 3 are located on the surface of the “head domains” of both pUL77-u and pUL77-l (Fig. 8B and D), where no interactions with other capsid proteins of the same subunit were detected.

**Fig 9:**
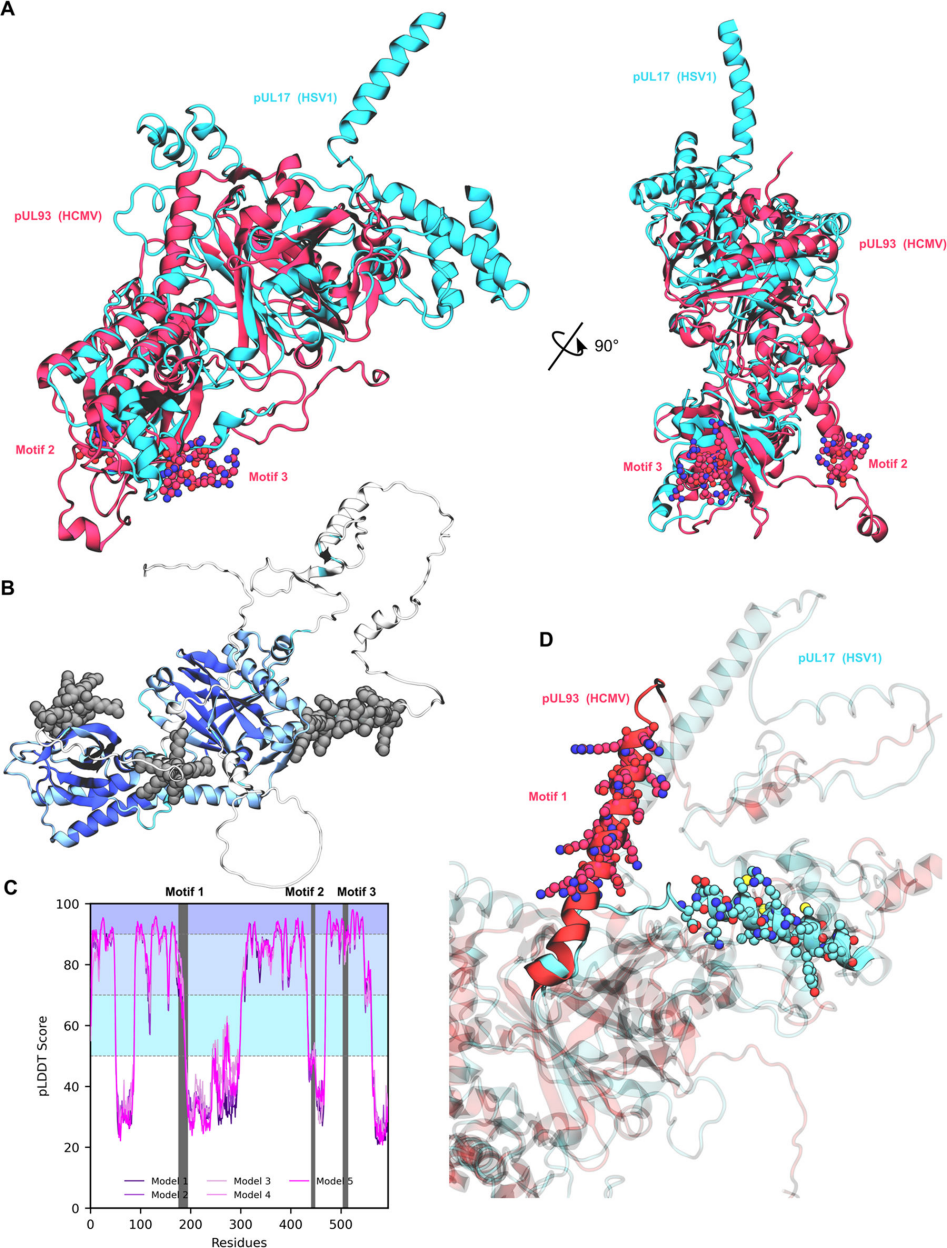
Structural comparison between HCMV pUL93 and its HSV-1 ortholog pUL17. (**A**) Structural superimposition of HCMV pUL93 (red, PDB ID 8TEP) and HSV-1 pUL17 (cyan, PDB ID 6GCR). (**B**) Predicted structure of HCMV pUL93 obtained by AF2, highlighting in gray motifs 1, 2, and 3. Secondary structure is colored according to the pLDDT score shown in (**C**). (**D**) Zoom of the predicted motif 1 region obtained from AF2 pUL93 model (red) superimposed on the resolved HSV1 pUL17 ortholog (cyan). For the sake of readability, pUL93 motif 1 and its corresponding sequence in HSV-1 are highlighted.

Likewise, we identified NLS 2 and 3 in the pUL93 resolved structure (Fig. 8E). While NLS 2 is located in the tail domain of pUL93 with no close contact with other capsid protein of the same subunit, NLS 3 was located at the interface between pUL77-l N-terminal domain and one of the five MCP. pUL93 NLS 3 is also rich in cationic residues (e.g., Arg507, Arg510, and Arg513) that are involved in attractive electrostatic interactions with surrounding electronegative of pUL77-l or MCP residues (e.g., Gln476, Gln543, and Glu547, see Fig. 8F). Since pUL93 NLS 1 was not experimentally resolved, we tried to decipher potential location using the HSV1 pUL17 (i.e., pUL93 ortholog) resolved structure (25), as well as AlphaFold2 predicted structure. It is important to note that the HSV-1 capsid structure determination was performed in a different configuration from that performed for HCMV, so the 3D structure observed may differ significantly depending solely on the protein state. Thus, the comparison of certain domains of the two homologs may be limited. We first aligned the resolved structure of HCMV pUL93 and HSV-1 pUL17 structures, for which relatively small structural differences were observed (root mean square deviation [RMSD] = 3.9 Å, Fig. 9A) in spite of a very poor identity score (9.44%). For instance, the front-barrel and back-barrel structure was conserved in pUL93. Interestingly, the region of pUL93 NLS 2 was not resolved in pUL17 ortholog, suggesting a configuration-dependent role. Unfortunately, the predicted location of pUL93 NLS 1 in the AlphaFold model exhibited a poor confidence score, precluding its use to hypothesize its potential role in, e.g., protein-protein interactions (Fig. 9B and C). However, the corresponding region was resolved in HSV1 pUL17, also adopting a kinked helix structure likely owing to the presence of Pro155. AF2 predicted a continuous α-helix structure containing a motif, even though this should be considered carefully owing to the poor pLDDT score (prediction local distance difference test: per-residue measure of local confidence) for this region. Moreover, the absence of a resolved structure from the HCMV cryo-EM structure suggests that such a domain is flexible.

## DISCUSSION

HCMV is a major public health problem by increasing morbidity and mortality in populations such as immunocompromised patients and neonates. The terminase complex was shown as a target of choice for the development of treatment against HCMV infections (5). So far, pUL77 and pUL93 have been mostly described as required structural partner proteins of the terminase complex for the genome encapsidation step (15, 26). However, the functional domains of these proteins have not been identified yet. Nevertheless, the HSV-1 homologs of pUL77 and pUL93, respectively, pUL25 and pUL17, have been more studied. It has been shown that pUL17 is required for capsid localization in the DNA replication compartments in the nuclei of infected cells, where cleavage and packaging of the viral genome take place (27, 28). Furthermore, pUL17 interacts with pUL25 to form the CVSC found on capsids’ vertices (17, 29). Currently, we know that HSV-1 CVSC is linked to capsid stability and core exit (30, 31). Moreover, Huet et al. demonstrated that pUL17 anchors the terminase complex to the capsid and that its interaction with pUL25 at the portal allows retaining the viral genome (32). In relation to this, pUL77 and its homolog pUL25 form the cap of the portal, which seals the capsid (24, 32). The functional motifs described in our study could therefore be involved in the sealing function.

Our results suggest that pUL77 and pUL93 are conserved proteins among HCMV reference strains and clinical isolates with average identities of 98.6% and 99.2%, respectively. We were able to identify the variable and conserved regions of pUL77 and pUL93 thanks to sequence alignment and comparison with other herpesvirus homologs. These results were also confirmed by means of polymorphism analysis for both proteins. Regarding pUL77, the only variable region was identified as a rich polymorphism domain in which the repetitive motif _145_PSDAVAPSDAVA_156_ is deleted. This suggests that this motif has no functional role for pUL77 but might be involved in favoring protein flexibility during complex formation. The first one hundred amino acids (1 to 92) of pUL77 have been structurally assigned to a coiled-coil motif, which was described as essential for pUL77 homodimerization and supramolecular interactions with MCP (15). These residues also belong to the capsid-binding domain that binds pUL48-u/l and pUL93, thanks to electrostatic interactions, and contributes to the CVSC helix bundle (24). However, our results surprisingly indicated that this region is not highly conserved, suggesting that the structural conformation of this region is more important than its level of conservation. After deletion of the pUL77 _522_DPAVTLSQLFPGLALLAV_539_ motif, the HCMV-BAC mutant did not replicate in MRC-5 cells. By 3D modeling, we observed that this motif was forming an alpha helix in the core of the protein. These results suggest that this motif is highly important for protein function and viral replication. This motif could therefore be involved in interactions with other proteins, such as pUL48, which are known to interact with pUL77 (13, 20). We can hypothesize that pUL77 (522-539) may also be involved in the protein-protein interactions between portal vertex subunits. For pUL93, three variable regions were identified for their rich amino acid polymorphism, while only seven small regions were described as highly conserved. This suggests that these seven conserved regions (I to VII) located in both N- and C-terminal might have relevant functional roles for pUL93 activity.

For each protein, two new amino acid mutations were identified in HCMV strains emerging in patients receiving letermovir prophylaxis: R43C and A161T for pUL77; E73G and R206H for pUL93. We built the recombinant mutants *in vitro,* for which no effect on viral growth nor LTV resistance was observed while considering standalone mutations, suggesting that they are simple polymorphisms, not related to the use of antivirals. In addition, clinical records showed that pUL56 mutations with a high level of resistance to LTV in patients are associated with these pUL77 and pUL93 mutations. Recombinant viruses with combined mutations were thus produced, and LTV EC_50_ was assessed. The results reported that the combination of these mutations with pUL56 V236M and C325Y mutations does not modify the LTV EC_50_s, similar to those obtained by Chou et al*.* in 2015 for pUL56 mutants (7, 8). However, the addition of the *UL77* R43C mutation to the *UL56* C325F mutation significantly attenuated the level of letermovir resistance. Absence of resistance mutations in pUL77 and pUL93 is supplementary proof that allows us to exclude interaction of LTV with the pUL77/pUL93 complex. Nevertheless, more patients with refractory infection under LTV prophylaxis must be explored to confirm our hypothesis. However, our results show that at least pUL77 would be in close contact with pUL56 and could modulate the mechanism of action of LTV.

We also investigated potential functional regions of pUL77 and pUL93 from the literature. Two putative NLSs have been reported for each protein, but those for pUL93 have never been studied (16, 21). We here showed that pUL77 NLS2* (_225_QPRWKRV_231_) is essential for HCMV viral replication but not for nuclear entry of pUL77. All these observations are in line with structural investigation since NLS1 is essential to (i) maintain C-terminal helices of pUL48-u/l thanks to strong electrostatic interactions, (ii) favor the helix-coil-helix configuration of at least pUL77-u but also likely pUL77-l, even though the flexible domain bridging N-terminal helices of pUL77-l has not been resolved yet. pUL77 NLS2* may play a role in capsid formation since NLS2* is located at the surface of the pUL77 head domain and might interact with the pUL77 protein of other subunits. Interestingly, evaluation of the pUL77 cellular localization by confocal microscopy showed that the deletion of these NLSs has no impact on nuclear translocation, in agreement with observations made by Köppen-Rung et al*.* (16). Altogether, these regions cannot be described as NLS domains, but they remain of particular importance for viral replication, suggesting a central functional role. This observation is in line with the AI-based structural study of the specific capsid top component published in 2024, in which pUL77 _43_RVRKRYLRQ_55_ is described as essential for anchoring the protein in the capsid (24).

Likewise, reported potential NLSs of pUL93 from the literature remain disappointing. Deletion of NLS1 (_177_KRDRQHLATTTNHRRR_193_) was associated with a slightly weaker nuclear translocation and a non-significant reduction in viral fitness. Deletion of pUL93 NLS2 (_442_RARRQ_447_) significantly impacted nuclear membrane crossing events. For these mutants, the decrease in nuclear localization could be impacted by a new tertiary conformation of the protein, which makes it more difficult to switch to the cell nuclei. However, we here proposed a new putative NLS motif, the so-called NLS3 (_505_RDRRGRLRR_513_), whose deletion drastically impaired viral replication in MRC-5 cells and clearly impacted nuclear crossing events in HEK293T cells. Deletion of pUL93 NLS3 may also impact the protein-protein interactions with MCP since NLS3 is located at their interface.

To take our analysis a step further, we co-transfected the HCMV BAC into HEK293T cells to allow the production of all HCMV AD169 proteins. In this context, the nuclear crossover events of all our constructs were enhanced or rescued. As with pUL51, which is required for correct nuclear crossing of pUL56 and pUL89 (33), our results led us to hypothesize that pUL77 and pUL93 are dependent on other proteins linked to the terminase complex or capsid. What’s more, we know that pUL77 and pUL93 are dimeric proteins, so their heterodimers may have an impact on their nuclear crossover and may rescue the deletion of different NLS, as has been demonstrated for the ribosomal protein RpS3 and its chaperone Yar1 (34). Further analysis is therefore required to better understand these mechanisms.

Finally, in our study, the 3D structure provides precise information on the conformation and atomic interactions of the protein, which is essential for understanding its biological function. However, sequence analysis allows the identification of conserved domains between different homologous proteins, which can reveal crucial functional regions, active sites, or interaction interfaces that are not always evident from the 3D structure alone. Furthermore, sequence-identified conserved domains can guide the functional interpretation of structure, help annotate poorly resolved or flexible regions, and facilitate evolutionary comparison between related proteins. The combination of the two approaches—structural and sequential—offers a more complete understanding of the function, evolution, and therapeutic potential of viral proteins.

According to the presented data, our study provides a better understanding of pUL77 and pUL93 structure and function. Our results suggested that the tertiary structure of these proteins is more important than their amino acid composition for their function. In addition, we have identified a new functional motif in pUL93 that is significantly linked to the nuclear localization of the protein and provided new data on the other two found by bioinformatics analysis in a previous study (21). Moreover, we demonstrated that nuclear crossing events of the two proteins, pUL77 and pUL93, were also linked to the presence of other viral components. This study also suggests that at least pUL77 could be an additional target for letermovir. Indeed, pUL77 CCM could be involved in modulating resistance to LTV by interacting with pUL56 and pUL89, as it was shown (16). Moreover, pUL77 and pUL93 could be of interest for the development of future therapies by inhibiting their functional domains that we described here through the development of antivirals or inhibitory peptides, as previously demonstrated for pUL51 and the NEC proteins (35, 36).

## MATERIALS AND METHODS

### Determination of conserved domains

For both proteins pUL77 and pUL93, the amino acid sequences of reference strain AD169 were aligned with the sequences of 17 homologous proteins from other herpesviruses, as described in Tables S1 and S2. Alignments were performed with the Clustal Omega (Ω) multiple sequence alignment tool, provided by the EMBL-EBI bioinformatics web tools and programmatic tools framework (37–39). To illustrate the degree of amino acid conservation, “*” was used to indicate a highly conserved amino acid; “:” was used to indicate a site belonging to a group exhibiting strong similarity; and “.” was used to indicate a site belonging to a group exhibiting weak similarity.

### Structural investigations and prediction of unresolved regions of pUL93 and pUL77

FASTA amino acid sequences of pUL93 and pUL77 were submitted to AlphaFold artificial intelligence software to predict protein structure (see Tables S1 and S2). Sequences were also submitted to the Expasy PROSITE Database (SIB Swiss Institute of Bioinformatics) to find relevant motifs in pUL77, pUL93, and their respective homologs in HSV-1, pUL25, and pUL17. Structural investigations were also conducted using the cryo-EM resolved structure of HCMV portal vertex adopting the virion configuration 1 (PDB ID: 8TEP) (24). In order to compare, the cryo-EM resolved structure of the HSV-1 capsid associated with tegument protein was also used (PDB ID: 6CGR) (25). Structural analyses and visualization, as well as rendering, were performed using the VMD 1.9.4α (Visual Molecular Dynamics) software (25).

### Cells, bacterial strains, and HCMV strains

MRC-5 human fibroblasts (bioMérieux, Craponne, France) were grown in minimal essential medium (MEM) containing 10% fetal bovine serum (FBS) with antimicrobials. HEK-293T (kindly provided by Gaëtan Ligat, Infinity Lab, Toulouse) were harvested in Dulbecco’s modified Eagle medium (DMEM) supplemented with 10% FBS, 2 mM of L-glutamine, and antimicrobials. All cells were placed at 37°C in 5% CO_2_. For BAC mutagenesis, *Escherichia coli* strain GS1783 was used (40). The HCMV-BAC (bacterial artificial chromosome containing the genome of the CMV laboratory strain AD169) contained a GFP gene in the unique short region and was derived from the parental strain pHB5, the BAC-cloned genome of HCMV laboratory strain AD169 (41). For the viral strains, five reference strains—AD169 (ATCC VR-538), Davis (ATCC VR-807), Towne (ATCC VR-977), Merlin, and Toledo—45 HCMV strain sequences from GenBank and 43 HCMV clinical isolate sequences from samples collected in various hospitals in France for the National Reference Center for Herpesviruses were studied. Clinical samples came from congenitally HCMV-infected neonates or HCMV-infected transplant patients. Twelve of the 43 patients were HSC recipients who had received LTV prophylaxis.

### Antiviral compound

Letermovir was purchased from MedChemExpress (HY-15233), reconstituted in 10 mM dimethyl sulfoxide (DMSO), and stored at −80°C. For antiviral assays, LMV was diluted in cell medium to a final concentration of 40 nM before making ½ serial dilutions down to 1.25 nM. For resistant strains, additional antiviral dilutions were made, ranging from 40 µM to 10 nM with a final DMSO concentration below 0.1%.

### Amplification and sequencing of the *UL77* and the *UL93* genes from reference strains and isolates

Complete genes were amplified after DNA extraction (E-Mag, bioMérieux, Craponne, France) from whole blood samples or from isolates by nested PCR using external and internal primers (Table S4) as described (42). Purified internal PCR products were sequenced (Applied Biosystems, Villebon-sur-Yvette, France) with sequencing primers designed using Geneious 9.1.8 software. Primers' specificity was assessed by alignment with GenBank strains. Sequencing results were analyzed using Geneious 9.1.8 software for comparison with AD169 gene sequences.

### Heatmap construction

The heat maps provide a data matrix where staining gives an overview of mutational differences between strains. They were generated using Excel spreadsheets (Microsoft, Washington, USA). Column width was set at 7 and row height at 0.17. Binary heat maps were plotted to show mutations in blue and the absence of mutations in white.

### Bacterial artificial chromosome mutagenesis

As described (40), to identify crucial amino acids involved in the identified putative motif, highly conserved residues were replaced by an alanine by “*en passant*” mutagenesis, using a two-step markerless red recombination system for BAC mutagenesis in *E. coli* strain GS1783 (40). Single mutations were introduced into an EGFP-expressing HCMV BAC (AD169 backbone), producing several mutants. Used primers are described in Table S5. For the pUL77 mutNLS1 construct, the amino acids were mutated in such a way as not to modify the pUL76 sequence. For other mutants, all regions of interest were deleted. For the pUL77 NLS2, three mutants (NLS2#, NLS2*, and NLS2) were constructed to evaluate the binary character of the region as described (16). Mutations were combined to assess a potential synergistic effect (Fig. 5B). SANGER sequencing prior to transfection for each recombinant virus confirmed the presence of the mutation/deletion. To ensure that the BAC backbone did not contain any other mutations that could have a negative impact on viral replication, we conducted NGS sequencing on both the original BAC and the mutants. The mutations/deletions were found in all mutant BAC sequences, while other SNPs were detected in genes that aren't essential for viral replication and represent less than 30% of the sequences, both in the original BAC and the mutants (11).

### Transient transfection

HCMV recombinant BACs were purified using the NucleoBond Xtra Midi system (Macherey-Nagel, Düren, Germany) following the manufacturer’s instructions. To reconstitute virus mutants, purified recombinant BACs were transfected into MRC-5 cells (bioMérieux, France) with Transfast liposomal reagent (Promega, USA) following the manufacturer’s instructions (43). The presence of mutations in the *UL77* and *UL93* genes of each recombinant virus in culture was confirmed by sequencing after extraction of viral DNA from each strain according to Hirt’s procedure (44).

### Plaque assays and growth curve experiments

Assessment of the impact of each mutation on viral fitness was carried out as described previously. We inoculated recombinant and HCMV-BAC AD169 WT strains into 48-well MRC-5 culture with a multiplicity of infection of 0.01. From day 1 to day 7 after inoculation, the number of fluorescent foci with cytopathic effect was counted to establish viral growth curves for each recombinant. The curves are the mean of three independent replicates, and the same for plaque assay results. For plaque assays, EC_50_s were calculated with the free EC_50_ calculator tool (https://www.aatbio.com/tools/ic50-calculator) (45). For statistical analysis, the Mann-Whitney test was applied (*P < 0.05, **P < 0.01, ***P < 0.001) using GraphPad Prism 8.4.3.

### Cloning of NLS mutants and confocal microscopy

Oligonucleotide primers used for vector construction are listed in Table S6. NLS mutants of *UL77* and *UL93* were PCR amplified from HCMV-BAC construction with primers mCherry-*UL77* forward/*XbaI-UL77* reverse and mCherry-*UL93* forward/*XbaI-UL93* reverse for N conformation of the mCherry gene, respectively, to not inhibit their function as seen in Fig. 5A. The mCherry gene was PCR amplified from the strain MG1655 rpoS-mCherry (46) with the primers *EcoRI*-mCherry forward/77-mCherry reverse and *NheI*-mCherry forward/93-mCherry reverse according to the desired conformation. Assembling the PCR mix contained 100 ng of each obtained fragments. The PCR program was as follows: first denaturation at 98°C for 5 min, followed by 35 cycles of 10 s of denaturation at 98°C, 45 s of hybridization at 58°C, and 10 s of elongation with a final elongation at 72°C for 5 min. Digestion was performed with *EcoRI*/*XbaI* and *NheI*/*XbaI* for inserts and PCI-neo plasmid (Promega, USA) containing *UL77* and *UL93*, respectively. The mCherry gene was fused with the N-terminal part of the *UL77* and *UL93* genes. Ligation of inserts and vectors was done with T4 ligase (New England Biolabs) at 16°C overnight in a 3:1 ratio. Ligation products were transformed in the chemically competent DH5-α strain by heat shock (Mix and Go, Ozyme). Transformed bacteria were cultivated on LB agar medium containing kanamycin (25 µg/µL) and ampicillin (25 µg/µL). Colonies were screened with the following primers pCI-neo F/pCI-neo R. Plasmids were extracted with the Xtra-Midi kit (Macherey-Nagel, Düren, Germany) according to the manufacturer’s instructions, and 4 µg of plasmid DNA were transfected into HEK293T cells in 6-well plates using Viafect reagent at a 4:1 ratio according to the manufacturer’s instructions (Promega, USA). After 72 h, cells were transferred into Labteck II cell culture chambers and were incubated for an additional 24 h. Cells were washed once with phosphate-buffered saline and fixed with a glacial 7:3 ethanol/acetone mix. Nuclei were stained with 4′,6-diamidino-2-phenylindole (DAPI) (blue fluorescence), and actin filaments were stained with AlexaFluor 488 phalloidin (ThermoFisher Scientific, USA) (green fluorescence). After staining, samples were mounted in ExPert mounting medium (CellPath, StatLab, USA) and examined by fluorescence confocal microscopy with Zeiss LSM880 confocal laser scanning microscope (Carl Zeiss, Germany). The same experiment was done with GFP-HCMV-BAC co-transfection. Wheat Germ Agglutinin Alexa Fluor 647 Conjugate was used to stain cell membranes (ThermoFisher Scientific, USA) (far-red fluorescence).

### Western blot analysis

Transfected HEK293T cells were lysed using RIPA buffer, and 50 µg of protein extracts were denatured with 2% β-mercaptoethanol mix and reduced at 70°C for 10 min. Proteins were characterized by SDS-PAGE and StainFree analysis (BioRad). Immunostaining was performed with a rabbit recombinant anti-mCherry monoclonal antibody (1:1,000) (#EPR20579, Abcam, USA) with an HRP goat anti-rabbit polyclonal antibody (1:5,000) (#ab97200, Abcam, USA) and an anti-cytomegalovirus IE1/IE2 mouse antibody (1:1,000) (#ab53495, Abcam, USA) with an HRP polyclonal goat anti-mouse IgG (1:1,000) (#ab6728, Abcam, USA). HRP anti-actin antibody was used to mark actin as a control (1:1,000) (#EPR16769, Abcam, USA). After each hybridization, the membrane was washed three times with TBS-T. PVDF membrane was revealed using FUSION FX Spectra (Vilber Lourmat, France).

### Statistical analysis of microscopy and western blot

To assess the reproducibility and truthfulness of our results, a minimum of three independent replicates of transfection for microscopy were done at different times. For each replicate, a minimum of 15 photos were taken at magnification ×63 to assess the percentage of mCherry expression in cell nuclei. Images were analyzed with the QuPath 0.5.0 platform (47) and ImageJ software (48).

For all these data, a statistical *t*-test was applied using GraphPad Prism 8.4.3: *P < 0.05, **P < 0.01, ***P < 0.001.

## Data Availability

The data that support the findings of this study are available from the corresponding author, C. Gourin or S. Hantz, upon reasonable request.

## SUPPLEMENTAL MATERIAL

Tables S1 to S6 jvi.01173-25-s0001.docx Strains, primers, and summary of mutations found in patients.

ASM does not own the copyrights to Supplemental Material that may be linked to, or accessed through, an article. The authors have granted ASM a non-exclusive, world-wide license to publish the Supplemental Material files. Please contact the corresponding author directly for reuse.
