# Supplementary material for: Functional and structural insights into HCMV terminase accessory proteins pUL77 and pUL93
Source: J Virol. 2025 Sep 16;99(10):e01173-25. doi: 10.1128/jvi.01173-25 (PMC12548450; doi:10.1128/jvi.01173-25)
Supplement: Tables S1 to S6 — Strains, primers, and summary of mutations found in patients. [file jvi.01173-25-s0001.docx]

**Functional and Structural Insights into HCMV Terminase Accessory Proteins pUL77 and pUL93**

C. Gourin,^1^ F. Di Meo,^2,3^ C. Delmon,^1^ S. Alain^1,4^ and S. Hantz^1,4,*^

^1^Inserm, CHU Limoges, University of Limoges, RESINFIT, U1092, F-87000 Limoges, France;

^2^Inserm U1248 Pharmacology & Transplantation, Univ. Limoges, Limoges, France

^3^Inserm US042/CNRS UAR 2015 Integrative Biology Health Chemistry & Environment, Univ. Limoges, Limoges, France

^4^CHU Limoges, Laboratoire de Bactériologie-Virologie-Hygiène, National Reference Center for Herpesviruses (NRCHV), F-87000 Limoges, France

*Correspondence: [claire.gourin@unilim.fr](mailto:claire.gourin@unilim.fr); [sebastien.hantz@unilim.fr](mailto:sebastien.hantz@unilim.fr)

# Supporting Tables

Supporting Table S1. Herpesvirus sequences used for pUL77 analysis

| **Identification** | **Virus** | **Protein** | **Length** | **Accession number** |
| --- | --- | --- | --- | --- |
| PrV | Suid alphaherpesvirus 1 | pUL25 | 536 | >UXR72849.1 |
| BoHV-1 | Bovine alphaherpesvirus 1 | pUL25 | 598 | >CAB01601.1 |
| HSV-1 | Human herpesvirus 1 | pUL25 | 580 | >QAU10208.1 |
| GaHV-1 | Gallid alphaherpesvirus 1 | pUL25 | 594 | >BAA36558.1 |
| GaHV-2 | Gallid herpesvirus 2 | pUL25 | 583 | >AAF66760.1 |
| HSV-2 | Human herpesvirus 2 | pUL25 | 585 | >QAU11017.1 |
| GaHV-3 | Gallid alphaherpesvirus 3 | pUL25 | 582 | >QEY02232.1 |
| VZV | Human alphaherpesvirus 3 | pUL25 | 579 | >NP_040157.1 |
| CyCMV | Cynomolgous cytomegalovirus | Cy77 | 595 | >YP_004933842.1 |
| RCMV | Rat cytomegalovirus Maastricht | pR77 | 656 | >NP_064177.1 |
| RhCMV | Macacine betaherpesvirus 3 | rhUL77 | 595 | >AAZ80608.1 |
| MCMV | Murid betaherpesvirus 1 | Pyruvol decarboxylase | 628 | >YP_214080.1 |
| HCMV | Human betaherpesvirus 5 | pUL77 | 648 | >AAS48968.1 |
| HHV6-A | Human betaherpesvirus 6A | U50 | 555 | >AJA36264.1 |
| HHV6-B | Human betaherpesvirus 6B | U50 | 555 | >QRY06542.1 |
| HHV7 | Human betaherpesvirus 7 | U50 | 554 | >AAC40764.1 |
| EBV | Human gammaherpesvirus 4 | pUL25 | 570 | >YP_401703.1 |
| SaHV-2 | Saimiriine gammaherpesvirus 2 | Unnamed protein | 543 | >NP_040221.1 |

Supporting Table S2. Herpesvirus sequences used for pUL93 analysis

| **Identification** | **Virus** | **Protein** | **Length** | **Accession number** |
| --- | --- | --- | --- | --- |
| PrV | Suid alphaherpesvirus 1 | pUL17 | 599 | >UXR72858.1 |
| BoHV-1 | Bovine alphaherpesvirus 1 | pUL17 | 702 | >ALR87810.1 |
| HSV-1 | Human herpesvirus 1 | pUL17 | 703 | >QAU10275.1 |
| GaHV-1 | Gallid alphaherpesvirus 1 | pUL17 | 717 | >AUT11980.1 |
| GaHV-2 | Gallid herpesvirus 2 | pUL17 | 743 | >AAF66752.1 |
| HSV-2 | Human herpesvirus 2 | pUL17 | 702 | >QAU11008.1 |
| GaHV-3 | Gallid alphaherpesvirus 3 | pUL17 | 722 | >BAB16525.1 |
| VZV | Human alphaherpesvirus 3 | pUL17 | 676 | >NP_040166.1 |
| CyCMV | Cynomolgous cytomegalovirus | Cy93 | 520 | >YP_004933867.1 |
| RCMV | Rat cytomegalovirus Maastricht | pR93 | 508 | >NP_064192.1 |
| RhCMV | Macacine betaherpesvirus 3 | rhUL93 | 521 | >AAZ80633.1 |
| MCMV | Murid betaherpesvirus 1 | E93 | 509 | >YP_007016490.1 |
| HCMV | Human betaherpesvirus 5 | pUL93 | 594 | >QHB20532.1 |
| HHV6-A | Human betaherpesvirus 6A | U64 | 442 | >NP_042957.1 |
| HHV6-B | Human betaherpesvirus 6B | U64 | 442 | >QRY06555.1 |
| HHV7 | Human betaherpesvirus 7 | U64 | 439 | >AAC40778.1 |
| EBV | Human gammaherpesvirus 4 | pUL17 | 507 | >YP_401692.1 |
| SaHV-2 | Saimiriine gammaherpesvirus 2 | Unnamed protein | 441 | >NP_040234.1 |

Supporting Table S3. Overview of clinical mutations in pUL56, pUL77 and pUL93.

| **LTV treated patient number** | **Gene mutations** | | |
| --- | --- | --- | --- |
|  | ***UL56*** | ***UL77*** | ***UL93*** |
| 1 | C325Y | - | - |
| 2 | V236M | - | E73G, R206H |
| 3 | - | - | - |
| 4 | C325Y | - | - |
| 5 | C325F | R43C | - |
| 6 | C325F | - | - |
| 7 | C325Y | - | - |
| 8 | - | - | - |
| 9 | C325Y | - | - |
| 10 | L257I | - | - |
| 11 | C325Y | R43C,A161T | - |
| 12 | C325Y | - | - |

Supporting Table S4. Primers used for amplification, screening and sequencing of UL56, UL77 and UL93

| **Primer name** | **5’ 🡪 3’ sequence** | **Tm** |
| --- | --- | --- |
| ***UL56* extern 1** | GTTGGAATAATCGTCGGGA | 54.7 |
| ***UL56* extern 2** | CGCGTCGCTGATGGACAAGT | 63.4 |
| ***UL56* intern 1** | GCGAGTTATTTGTGCACCG | 57.7 |
| ***UL56* intern 2** | GATATTACGTTCAAAGCGAA | 51.8 |
| **UL56 1** | ATAGGCTTTTGAGGGCCGA | 58.7 |
| **UL56 2** | CCGTCAAGCCAGTCCGAT | 59.4 |
| **UL56 3** | CCTCTGACTGTGAGTACTGCTGT | 61.6 |
| **UL56 4** | GCATATTCTCGAGGAGATTCG | 56.1 |
| **UL56 5** | TCCTTCTGCAGCACGTTTAG | 58.2 |
| **UL56 6** | ATCCCCTCTCTCACAATGTG | 56.6 |
| ***UL77* extern 1** | GCGGCATCGTGTATTGCTAC | 59.8 |
| ***UL77* extern 2** | ATCGGCCGACATAGAGTAGC | 59.1 |
| ***UL77* intern 1** | TTGATTTTCTGCGTCTGCCG | 59.5 |
| ***UL77* intern 2** | CCGAACGAATATACCCTCTCCG | 60.1 |
| ***UL77* 1F** | GTTGAACCAGCAGTGCGAG | 59.4 |
| **UL77 1R** | CCAGGTAGAAGAAGCACCGG | 60.1 |
| **UL77 2F** | GGTGTATCCGACGTACGACT | 59.0 |
| **UL77 2R** | CTTCCCAAGCGGCAATCTCA | 60.7 |
| **UL77 3F** | GTCTTTGTGCACGAACAGCA | 59.6 |
| **UL77 3R** | TACCACGGCCCGATGTAGTA | 60.1 |
| ***UL93* extern 1** | GACGTCAACATCATCAGCGC | 60.0 |
| ***UL93* extern 2** | TGCTGGTCACCTGGCTAAAG | 60.0 |
| ***UL93* intern 1** | TACGACGCGTGTGTCATCAA | 60.0 |
| ***UL93* intern 2** | CTTGATAACGGCCGTTGCAG | 59.9 |
| **UL93 1F** | TTAGAGAAAGAGCAGCGCGT | 59.8 |
| ***UL93* 1R** | CGCCGACGGTGATTCGTG | 61.5 |
| **UL93 2F** | CAGACCTCATGTACGCCACC | 60.5 |
| **UL93 2R** | GCGCCAAAAGGAATTGACGT | 60.0 |
| **UL93 3F** | CGTAGCTGTATTATTAAGGCGCT | 58.8 |
| **UL93 3R** | CGAGAGCCAACGTCGCAG | 61.2 |

Supporting Table S5. Primers used for HCMV-BAC mutant construction in UL56, UL77 and UL93

| **Primer name** | **5’  3’ sequence** |
| --- | --- |
| ***UL56* C325F For** | GGGCATCACCATCCAGCAGCTAAATGTGTATCACCAGCTGTTCCGGGCGCTCATGAACGGCATTAGGGATAACAGGGTAAT |
| ***UL56* C325F Rev** | CCCCGTACAGATGGCGACTGATGCCGTTCAGCGCCCGGAACAGCTGGTGATACACATTTAGCCAGTGTTACAACCAA |
| ***UL56* C325Y For** | CCCCGTACAGATGGCGACTGATGCCGTTCATGAGCGCCCGGTACAGCTGGTGATACACATTTAGCCAGTGTTACAACCAATTAACC |
| ***UL56* C325Y Rev** | GGGCATCACCATCCAGCAGCTAAATGTGTATCACCAGCTGTACCGGGCGCTCATGAACGGCATTAGGGATAACAGGGTAATCGATT |
| ***UL56* V236M For** | CCACCGTCCGTCAAGCCAGTCCGATGTGAATATCCAGACGATGGAGCAGGACCTGCTGGACCTTAGGGATAACAGGGTAATCGATT |
| ***UL56* V236M Rev** | AGTGGGGGATGCGCGTTGTCAGGTCCAGCAGGTCCTGCTCCATCGTCTGGATATTCACATCGGGCCAGTGTTACAACCAATTAACC |
| ***UL77* R43C For** | CGTGCTTCGGCGGTTGCTGGAGGACGCGGCGGTGACAATGTGCGGCGGGGGCTGGCGCGAGGATAGGGATAACAGGGTAATCGATT |
| ***UL77* R43C Rev** | GCACCCGGTCCATGAGCACGTCCTCGCGCCAGCCCCCGCCGCACATTGTCACCGCCGCGTCCTGCCAGTGTTACAACCAATTAACC |
| ***UL77* A161T For** | CGTGGTCGCCCCGTCTGACGCGGTCGCCGCGTCAGCGGCCACCGGTGCTTCTTCTACCTGGCTTAGGGATAACAGGGTAATCGATT |
| ***UL77* A161T Rev** | GCCGCTCGGCGCACTGCGCCAGCCAGGTAGAAGAAGCACCGGTGGCCGCTGACGCGGCGACCGGCCAGTGTTACAACCAATTAACC |
| ***UL93* E73G For** | GGGTGGAGATGAGGACGACGCACCGGCCTCCGACGACGCCGGGAACGCCGTGGGCGGCGATCGTAGGGATAACAGGGTAATCGATT |
| ***UL93* E73G Rev** | GCCGCTCGCGGTCAAAAGCGCGATCGCCGCCCACGGCGTTCCCGGCGTCGTCGGAGGCCGGTGGCCAGTGTTACAACCAATTAACC |
| ***UL93* R206H For** | GCGGGGCGGACTGCGTAACAATTTAGACAATGGGTCGGATCACCGATTGCCCGAAACGGCTATTAGGGATAACAGGGTAATCGATT |
| ***UL93* R206H Rev** | CGGCCGTCTCCAAAGAAGCCATAGCCGTTTCGGGCAATCGGTGATCCGACCCATTGTCTAAATGCCAGTGTTACAACCAATTAACC |
| ***UL77* NLS 1 rev** | AATGCGCGGCGGGGGCTGGCGCGAGGACGTGCTCATGGACCTGGAGCACAGACTGTCTCAGCTTCTGGAGCTCAGGGATCTGGGTCATAGGGATAACAGGGTAATCGATT |
| ***UL77* NLS 1 for** | CGCAGTAAGTCTGCACCCTGTGACCCAGATCCCTGAGCTCCAGAAGCTGAGACAGTCTGTGCTCCAGGTCCATGAGCACGTCCTCGCGCCAGTGTTACAACCAATTAACC |
| ***UL77* NLS 2 for#** | CACCATGTTCGAGAATGCCTCTACTTGGACTTTCTCCTTTCAACCACGGTGGAAACGAGTTAGGGATAACAGGGTAATCGATT |
| ***UL77* NLS 2 rev#** | CCATCTGCGCCAGATGGTACACTCGTTTCCACCGTGGTTGAAAGGAGAAAGTCCAAGTAGGCCAGTGTTACAACCAATTAACC |
| ***UL77* NLS 2 for*** | TATCTGGTACTATCGGCTCAAGCGGGGGTTGTACACGCAATACCATCTGGCGCAGATGGATAGGGATAACAGGGTAATCGATT |
| ***UL77* NLS 2 rev*** | CCTGCGAAATGGAAAAGTTGTCCATCTGCGCCAGATGGTATTGCGTGTACAACCCCCGCTGCCAGTGTTACAACCAATTAACC |
| ***UL77* NLS 2 for** | CGAGAATGCCTCTACTTGGACTTTCTCCTTTGGTATCTGGTACCATCTGGCGCAGATGGATAGGGATAACAGGGTAATCGATT |
| ***UL77* NLS 2 rev** | CCTGCGAAATGGAAAAGTTGTCCATCTGCGCCAGATGGTACCAGATACCAAAGGAGAAAGGCCAGTGTTACAACCAATTAACC |
| ***UL77* Δ522-539 for** | TCGGGCCATGGTACGCGCGCACCGAGAGCGTGCGCAGCGGTTGGGATCCCTCACGTCGCGTAGGGATAACAGGGTAATCGATT |
| ***UL77* Δ522-539 rev** | CCGCTGCGCACGCTCTCGGTGCGCGCGTACCATGGCCCGATGTAGTACTGCACGAGAAACGCCAGTGTTACAACCAATTAACC |
| ***UL93* NLS1 for** | TTTAGCGCGTGACCTGTTACGAGAAGAAATGGAAGCGAATGGCGGACTGCGTAATAACTTTAGGGATAACAGGGTAATCGATT |
| ***UL93* NLS 1 rev** | GGCGATCCGACCCATTGTCTAAGTTATTACGCAGTCCGCCATTCGCTTCCATTTCTTCTCGCCAGTGTTACAACCAATTAACC |
| ***UL93* NLS 2 for** | AACGCCTCGTCTGAGCCGGGGTGCCGCCGCTGCCGCTCAGCGCGAGCGCTCGGCGCCCAATAGGGATAACAGGGTAATCGATT |
| ***UL93* NLS 2 rev** | CGAAAAGCAGCTCCTGCGGTTTGGGCGCCGAGCGCTCGCGCTGAGCGGCAGCGGCGGCACGCCAGTGTTACAACCAATTAACC |
| ***UL93* NLS 3 for** | CGCGCGGCGCGTTGTCCTCGCACCGGGCTTTGGATCGTCTGGCTCTCGCAGCCCGAGGTTAGGGATAACAGGGTAATCGATT |
| ***UL93* NLS 3 rev** | GCGTGACGTACAGCACGCACACCTCGGGCTGCGAGAGCCAGACGATCCAAAGCCCGGTGCGCCAGTGTTACAACCAATTAACC |

Supporting Table S6. Primers used for cloning of pUL77 and pUL93 in pCI-neo with mCherry

| **Conformation** | **Primer name** | **5’ 🡪 3’ sequence** |
| --- | --- | --- |
| N | mCherry-*UL77* forward | CGAGCTGTACAAG ATGAGTCTGTTGCACACCTTTTG |
| N | *XbaI*-*UL77* reverse | **CTAGTCTAGA** TTACAACACCGCCACGCTCGGAAG |
| N | *EcoRI*-mCherry forward | **AATTGAATTC** ATGGTGAGCAAGGGCGAGG |
| N | *UL77*-mCherry reverse | GTGCAACAGACTCAT CTTGTACAGCTCGTCCATGC |
| N | mCherry-*UL93* forward | CGAGCTGTACAAG ATGGAAACGCACCTGTATTC |
| N | *XbaI*-*UL93* reverse | **CTAGTCTAGA** CTAAAGATCGTCGAACGGCAAG |
| N | *NheI*-mCherry forward | **CTAGGCTAGC** ATGGTGAGCAAGGGCGAGG |
| N | *UL93*-mCherry reverse | CAGGTGCGTTTCCAT CTTGTACAGCTCGTCCATGC |
|  | pCI-neo Forward | TCTCTCCACAGGTGTCCACT |
|  | pCI-neo Reverse | CACATCTCCCCCTGAACCTG |
| **Bolded nucleotides:** restriction *NheI*, *XbaI* or *EcoRI* site with additional bases for correct cleavage | | |
